# Including the patient voice in the development and implementation of patient‐reported outcomes in cancer clinical trials

**DOI:** 10.1111/hex.12997

**Published:** 2019-11-13

**Authors:** Bonnie Addario, Jan Geissler, Marcia K. Horn, Linda U. Krebs, Deborah Maskens, Kathy Oliver, Ananda Plate, Erin Schwartz, Nicole Willmarth

**Affiliations:** ^1^ GO_2_ Foundation for Lung Cancer San Carlos CA Washington DC USA; ^2^ Patvocates Riemerling Germany; ^3^ ICAN International Cancer Advocacy Network Phoenix AZ USA; ^4^ International Society of Nurses in Cancer Care Vancouver BC Canada; ^5^ International Kidney Cancer Coalition Guelph ON Canada; ^6^ International Brain Tumour Alliance Tadworth UK; ^7^ Myeloma Patients Europe Brussels Belgium; ^8^ The Max Foundation Seattle WA USA; ^9^ American Brain Tumor Association Chicago IL USA

**Keywords:** cancer, clinical decision making, patient advocacy, patient‐reported outcomes

## Abstract

**Context:**

Patient‐reported outcomes (PROs) are used in parallel with clinical evidence to inform decisions made by industry, clinicians, regulators, health technology assessment bodies and other health‐care decision‐makers. In addition, PRO data can also guide shared decision making and individual patient choice. Yet, the quality of many PROs in cancer clinical trials is suboptimal and requires improvement to add value to health care and policy decision making.

**Objective:**

To show how the integration of the patient and/or patient advocate at all stages of PRO development can help to realize the full potential of PROs.

**Methods:**

We examined the literature to show that the patient voice is often absent from the planning and implementation of PROs in cancer clinical trials. Good practice examples from the literature were combined with guideline recommendations, training or educational resources, and our own experience to create detailed practical steps for the inclusion of patients and/or patient advocates throughout PRO development.

**Results:**

Patient or patient advocates can play an active role in shaping PROs that are meaningful to the patient. They can contribute to content, choice of medium and implementation in a way that may support PRO completion and minimize missing data. Patients and their advocates can work to ensure PRO findings are disseminated appropriately in a way that is accessible to patients.

**Conclusion:**

This practical guidance aims to optimize PRO development and implementation in clinical trials, resulting in robust, relevant data that reflect the patient experience and that support decisions made by all stakeholders involved in research and health care.

## 1 INTRODUCTION

Patient‐reported outcomes (PROs) have been used in cancer clinical trials for over two decades.^1^ These evaluations, captured electronically or in person, in formats ranging from questionnaires to wearable devices, serve to provide a unique record of the patient's lived experience of a disease, its treatment and management, and the impact these may have on function and health‐related quality of life (HRQoL).^2^, ^3^ The inclusion of PROs as trial endpoints aims to ensure a comprehensive assessment of burden of disease and the impact of an intervention.^4^ In this way, PROs have established themselves as a central and indispensable component of the evidence evaluating medicines and can be used by clinicians, patients and policy makers to assess treatment choice, shape guidelines and enable regulatory and policy decisions based on the benefits and costs of treatment.^5^, ^6^ The inclusion of PRO data in clinical trials is supported by international guidelines, professional organizations and by regulatory and health technology assessment (HTA) bodies for measuring patient experiences that are not captured by conventional efficacy or adverse event data.^3^, ^6^, ^7^, ^8^, ^9^, ^10^ In practice, however, few PROs in pre‐approval oncology clinical trials are reported to meet the Food and Drug Administration (FDA) standards.^11^ Of the 40 treatments approved by the FDA Office of Hematology and Oncology Products between 2010 and 2014, only three (7.5%) were granted PRO labelling.^12^ Collection of PROs has been shown to be inconsistent in cancer clinical trials with less than a third of the recommended PRO‐related items included on average in study protocols in the UK.^13^ PROs are not always accepted in HTA review processes: one recent study of submissions across several European countries found that PROs were mentioned in the final decision in less than half and were not reviewed by the HTA body in 21%.^14^

Reasons cited for the poor quality of PROs in some cancer clinical trials include methodological, cultural and practical issues.^3^, ^15^ Choice and content of PRO measures are not always relevant to the aim of the study or may not reflect what is important to the patient.^4^, ^16^, ^17^ PROs are all too often dismissed as subjective in contrast to laboratory findings and as less important than survival endpoints.^11^, ^17^ In its guidance on PROs in regulatory submissions, the European Medicines Agency (EMA) refers to a range of issues including bias, timing of assessments and missing data.^3^ The practicalities of implementation may not be conducive to patients completing a PRO measure or to administrative staff supporting them in doing so.^4^ In some cases, compliance with completion of PRO measures is so poor that the data are not even analysed.^18^, ^19^ PRO reporting can lack transparency, failing to provide details such as the rationale for a PRO measure or the approach to missing data, hampering interpretation.^20^ Furthermore, PRO results may not be disseminated to the most relevant audiences or are not always reported in an accessible way.^4^, ^21^

As leaders of patient advocacy organizations, we believe that the failure of many PROs to fully reflect the patient perspective means that decision‐makers such as clinicians, industry representatives, regulators, policy makers, HTA bodies and patients themselves are missing an important piece of evidence that could potentially resolve uncertainties about value and aid decision making. We urgently need to integrate the patient voice throughout PRO development and implementation, rather than the more usual practice of the patient acting as a consultant at certain stages of PRO development.^16^ Incorporating the patient perspective will optimize the relevance of PROs in cancer clinical trials and support the delivery of patient‐centred care. We are not alone in this view: patients, clinicians, industry and regulatory bodies have shown their support for a collaborative approach to the development of PROs and corresponding measures.^7^, ^16^, ^22^, ^23^ This does not yet happen routinely in cancer clinical trials.^16^ How we might achieve such an approach and what benefits this may bring is the subject of this paper.

## 2 THE PATIENT AND PATIENT ADVOCATE CONTRIBUTION

**Figure 1:**
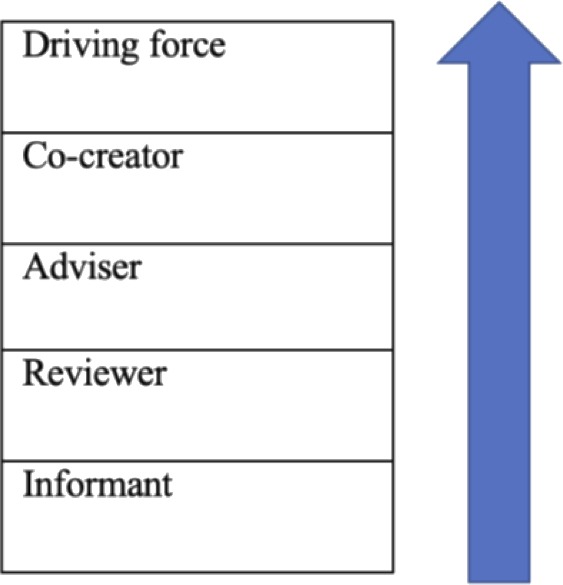
Levels of patient and patient advocate engagement (adapted from Wilson et al^25^)

In order for the patient voice to be integrated throughout the development, implementation and dissemination of a PRO, it needs to be included from the outset.^24^, ^25^ This enables a truly collaborative approach with patients, patient advocates and in some cases caregivers as patient partners, moving beyond traditional roles as consultants or providers of information and into roles as advisers, co‐creators and even drivers of the process, as shown in the model of the participation ladder (Figure 1).^24^, ^25^

### 2.1 PRO design and selection

Patients and patient advocates can play a central role in the design and selection of PROs. They bring to the process knowledge of the disease, symptoms and attributes of care with the greatest impact on patient lives. This patient perspective can be combined with the health‐care professional perspective to form a holistic disease model which provides a foundation for the subsequent selection or design of a PRO.

Choosing a study endpoint takes place early in the planning process and patients and patient advocates can contribute to the identification of PRO‐assessed endpoints that are meaningful to patients, such as pain or unmet medical needs. In a disease where quality of life can be a priority for patients, patient experiences and outcomes may well be considered at least as important as improvements in survival in shaping research priorities.^11^, ^26^ Cancer patient psychosocial well‐being and physical well‐being are not mutually exclusive: psychosocial factors, such as depression, have been shown to predict recovery and health status after surgery.^27^

Subsequently, the patient or patient advocate voice can be combined with the findings from a literature review and expert input in order to define a conceptual framework that will detail the rationale of the PRO and the specific concepts it aims to measure; for example, experience of pain, fatigue or HRQoL.^6^, ^7^, ^28^ It should also detail the intended population who will complete the measures.^12^, ^18^ Patients and patient advocates can ensure that the PRO concept is meaningful and relevant to the intended population and can contribute further by selecting domains for inclusion in the PRO that reflect the concept and ensure that the patient experience is captured fully.^6^, ^7^, ^29^ This may involve identifying domains that are missing from existing PROs: the social and emotional experiences of cancer patients, for example, are not always assessed.^30^, ^31^ Domains not relevant to the intended population may also be identified.

Using their familiarity with the patient experience, patients and their advocates can provide input into item selection for any existing or new measures, giving careful consideration to the symptoms and effects associated with a specific type of cancer. They should also take into account whether the study participants are starting treatment, receiving treatment or living with or beyond the disease.^21^

#### 2.1.1 Patient‐reported events

Patients and patient advocates might consider calling for the use of a PRO measure in a clinical trial to document both positive and negative effects experienced by the patient that may otherwise not be identified as disease‐ or treatment‐related. Discrepancies can exist between health‐care professional and patient perception of adverse events (AEs) associated with treatment^32^ and clinicians, unlike patients, are required to judge how likely an AE is to be treatment‐related. It is important that clinicians' judgement on the relevance of an AE does not include only AEs that can be clinically managed while AEs such as fatigue are not taken into account. A measure that records patient‐reported events has the potential to provide a comparison with clinician reports. Industry, HTA bodies, regulatory bodies or payers can assess PRO AE findings rather than relying on interpretation or selection by trial investigators or clinical experts.

One such tool has been developed. Based on the Common Terminology Criteria for Adverse Events (CTCAE), the PRO‐CTCAE allows patients to record their experience of AEs that do not require laboratory assessment, without clinician selection and interpretation.^33^, ^34^ Patient representatives were active participants throughout the development of this measure and a wider group of patients was involved in providing detailed feedback at several points in the process.^34^ Basch and colleagues have shown that patients are willing and able to complete the PRO‐CTCAE for the collection of adverse event recording and that minimal additional resources are required.^35^ Making the use of a PRO on AEs mandatory may provide a better assessment of the balance between the efficacy and toxicity of cancer treatments.^36^

Patients and patient advocates recognize the negative impact that a grade 1 or 2 AE, such as nausea or fatigue, can have on an individual's quality of life if it is persistent rather than transient and may suggest that PRO measures include duration as well as frequency.^37^, ^38^ This is particularly relevant in the cancer patient population where extended survival may mean that treatment is given over longer periods of time. Additionally, all grades of AE data (from mild AEs at grade 1 to severe AEs at grade 4) should be included in trial reports, not only AE data of grade 3 or above which can result in under‐reporting of toxicity.^36^, ^39^ Use of patient education tools provided by patient advocates is beneficial in supporting patient understanding of the differences between AE grades.^40^ Patients and patient advocates may also choose to add their voice to the call for standardized AE grading decision criteria.^40^ Finally, in discussion with their research partners on AEs, patients and patient advocates are able to support the necessity of disseminating full AE data so that individual patients can make informed choices. Some patients may be prepared to tolerate greater AEs than others based on age, stage in their patient journey and other factors.^41^ Patient‐reported AE data are increasingly accepted or solicited by regulatory and HTA bodies and can play a key role in their decision making.^42^ In the case of the non‐small‐cell lung cancer treatment crizotinib, for example, post‐marketing PRO data on symptom control and improved HRQoL resulted in a reversal of an earlier negative reimbursement decision by the German HTA body which was based on data only from the initial study endpoints.^42^

#### 2.1.2 Good practice examples of design and selection

Integration of the patient voice in the development of PROs used in cancer clinical trials does not appear to be commonplace to date.^15^ Emerging examples do exist, however, of patient and patient advocate involvement in the design or selection of PROs in the routine care setting and in patient surveys and registries that are engaging with key questions that are relevant to PRO use in clinical trials.^43^, ^44^, ^45^, ^46^

Patients and patient advocates can offer valuable advice on the challenging question of whether to use standardized pre‐existing PRO instruments, or to adapt or devise a new measure. Standardized PRO sets are recognized as enabling population comparisons, which are of value to comparative processes such as HTA assessment, in addition to making efficient use of resources (Table 1).^17^, ^43^ However, experience of a specific patient population may not be reflected in a standardized measure.^47^ Where a PRO measure does not reflect the patient experience in question, the choice is whether to adapt an existing measure with the same concept, to combine validated generic and disease‐specific measures in a trial or to develop a new measure.^47^, ^48^ An example of a PRO measure developed for a specific population is outlined in Table 2. Patients and patient advocates may consider whether the PRO aims to enable comparison with other PROs or rather to facilitate understanding of a specific group of patients by documenting their experience.

**Table 1:** Example of patient and patient advocate involvement in the development of a taxonomy of core domains and measures for national use in Canada^43^

PRO measures/domains	Core domains, subdimensions and measures to be identified for use in routine cancer care across Canada
Development partners	11 multidisciplinary health‐care professionals Five cancer survivors representing four national patient advocate organizations
Focus	Standardization of measures used, allowing national comparisons as well as improving individual care
Role of patients/patient advocates	Scoping review of literature Consensus process to select domains and PRO measures
Result	Formulation of a patient‐focussed taxonomy of 20 domains, related subdimensions and 45 self‐report measures that were considered relevant and feasible for collection

**Table 2:** Example showing role of patient and patient advocate input in the assessment of caregiver burden in glioblastoma (GBM)^44^

PRO measure	Survey tool used in an ongoing study which aims to assess the effect on caregiver burden of cognitive dysfunction in individuals with GBM
Development partners	Clinical researchers American Brain Tumor Association (ABTA) Caregivers of patients with GBM
Role of patients/patient advocates	ABTA was involved early and throughout the process Patient advocates and caregivers were active participants in shaping the domains of the PRO, developing the survey instrument and in defining the inclusion criteria ABTA representatives took part in focus groups with neuro‐oncologists to discuss findings based on a literature review
Result	The approach has enabled the generation of a GBM‐specific caregiver survey tool which is useful for understanding caregiver burden in this disease population

Registries are becoming increasingly important for decision‐makers. The EMA is currently overseeing an open consultation on the use of patient disease registries for regulatory purposes, which addresses challenges such as harmonization of methods and data sharing.^49^ Substantive patient or patient advocate involvement is evident within the context of disease registries. The Lung Cancer Registry, set up in 2016 by the Bonnie J Addario Lung Cancer Foundation working in collaboration with other patient and professional groups, works to involve patients directly in the collection of data in order to establish a direct communication network between patients and patient advocates, researchers, health‐care professionals, industry and policy makers.^45^ Registry patients were involved in this way in one recent study which examined patient‐reported toxicities and quality of life in lung cancer patients treated with immune checkpoint inhibitors.^46^ Crucially, patients from this group along with oncology providers and informal caregivers were interviewed to develop an item bank on toxicities, which formed part of the PRO measure used.^46^ These examples, along with the elements of PRO design and selection outlined above, show the potential of the contribution of patients and patient advocates to developing or choosing a PRO for use in a clinical trial that fully reflects the patient experience.

### 2.2 PRO implementation and administration

The patient and their advocates can also bring insights based on experience to guide practical decisions around PRO implementation and administration. They are well placed to collaborate on the production of a trial protocol which details how, when and by whom the PRO should be implemented, along with a record of the conceptual framework.^6^ Lack of such detail in the protocol may result in methodological inconsistencies between centres and how individuals implement or document findings. This can result in suboptimal data quality.^19^

#### 2.2.1 Missing data

Not all missing PRO data are avoidable but large amounts of missing PRO data can have implications for analysis: potentially biasing interpretation; compromising the validity of study findings; or resulting in treatments being denied PRO labelling by regulatory bodies.^12^, ^19^

The completion of PRO measures must not be burdensome for either well or unwell patients.^21^ Shorter and more reliable measures can improve response rates.^48^ To make completion of measures easier, patients and patient advocates can work towards keeping measures short, using accessible language and asking only questions that are pertinent to patient experience. Patient input into the debate around the choice of a measure or a balance between generic and disease‐specific measures can advise on the capacity of patients to complete the questionnaires proposed. In the same way, patient and patient advocate advice on setting the recall period would be grounded in their experience of what may be realistic for a patient at any given point. The patient representative can advise when PRO completion by proxy may be necessary, where the patient is very young, very elderly, critically ill or under emotional distress, for example, and can work to ensure guidelines on inclusion in the trial protocol are followed to minimize any bias this may introduce.^6^

The research value of PROs may not be fully understood by the patient. This may have a negative impact on the collection of PRO data.^19^ Some patient advocacy organizations offer educational support on this aspect in both the research and routine care setting, encouraging patients to complete the measures they are given; providing support to patients who are facing challenges in completion; and reassuring them that their responses will help them receive better care on the research study or in the clinic. Advocates can also prompt study participants to remind their health‐care professional if a PRO questionnaire they are expecting is not received.

#### 2.2.2 Linguistic and cultural input

Patients and patient advocates can make an informed contribution to the language used in questionnaires in order to ensure the use of plain language, the lack of which has been noted in some existing cancer PRO measures.^50^ Similarly, patients and patient advocates from each region or community where the measure will be used can help validate linguistic and cultural aspects a PRO measure in order to make it understandable and accessible to all members of the study population.^51^ Supporting the use of plain language in PRO measures and making sure terms are understood in the same way across different cultures, and health‐care frameworks may also increase compliance for patients with low literacy levels. Patients from different nationalities who collaborated on the development of a rheumatology PRO measure were also involved in translating that measure for their respective populations.^25^ Such involvement may facilitate wide geographical and cultural use of standardized PROs, enabling broad comparison of research findings as well as national and international population monitoring and policy development.^52^

#### 2.2.3 Achieving inclusivity

Importantly, patients and patient advocates from low‐ or middle‐income backgrounds or countries appear to be less frequently involved in PRO development. Some of the studies discussed in this paper have found the inclusion of people from a range of ethnic and socio‐economic backgrounds challenging.^24^, ^43^ One role of the patient advocate organization may be to advise on including patients from diverse cultural and economic backgrounds and of different ages in the development of PROs or, if that is not possible, to ensure that the draft PRO includes diverse voices in the validation process. The inclusion of diverse populations in clinical trials can also be problematic.^53^ If some groups of patients or cultural contexts are not represented at the design stage in a clinical trial, then any PROs used may not capture their experience, excluding the experience of that community and making findings less generalizable. Advocates, clinicians and trial sponsors should collaborate at an early planning stage to ensure as diverse a range of patients as possible is included in clinical trials.

#### 2.2.4 Medium, setting and technologies

Recognition is increasing that in order to improve data quality and adherence to data collection, PRO data need to be collected in ways beyond the standardized questionnaire format on cancer clinic days.^4^, ^54^ Indeed, electronic PROs are increasingly incorporated into clinical trials.^55^ Smartphones, tablets and computers allow prompt response from participants during a clinical trial and enable real‐time inclusion of PRO responses in patient–clinician consultations on site or remotely when an electronic PRO is linked to the hospital's information system.^54^ Qualitative interviews may be conducted over the Internet for patients for whom a face‐to‐face meeting is difficult. Wearable devices can record patients as they go about their daily lives. Patients and patient advocates can offer advice on the suitability of each medium for the study population, how often a PRO might be completed to understand the patient's day‐to‐day experience and whether the clinic, home or other setting is the most convenient or appropriate place to complete PRO measures. They can also advise on the likelihood of any training requirements for patients to enable use of new technologies. Table 3 shows a good practice example of patient and patient advocate integration in developing an electronic PRO in routine cancer care. Including the patient perspective to support the use of new technology in a population that may not be familiar with it may also be of great value in clinical trials. The same technologies that are used to support PRO completion may be used to facilitate greater patient numbers or more frequent input into the collaborative development of the PRO itself. 25 Smartphone or Internet feedback may enable greater numbers of patients and patient advocates to be involved in a PRO validation process, for example. In summary, the involvement of patients and patient advocates in PRO development, implementation and administration can itself be supported by new technologies and can facilitate and encourage patient completion of the PRO resulting in more comprehensive and robust PRO clinical trial data.

**Table 3:** Example showing advantages of the collaborative development of an electronic PRO^54^

PRO measure	Online questionnaire to report adverse events during cancer treatment in the routine care setting
Development partners	Project team: Researchers Lead oncology clinicians Health informatics experts Patients and patient advocates Wider stakeholder group: Clinical staff including nurses and physiotherapists Patient advocates Patients Administrators Researchers
Role of patients/patient advocates	Participation in shaping technical specification of system to suit the needs of both patients and clinicians Shared development of clinically based algorithms that sent immediate, automated tailored advice on managing any AEs Testing usability and functionality of the measure
Result	Development of electronic PRO enabling patients to report AE from home on PC, tablet or any web‐enabled device securely during cancer treatment and to receive prompt clinical advice. System evaluation is ongoing

### 2.3 Dissemination of PRO findings

PRO data can remain unpublished or may not reach the relevant stakeholder groups, including patients.^17^, ^21^ However, patients and patient advocates can use their patient community networks to support the dissemination of PRO findings. Patient advocates are a bridge between their patient constituencies and the clinical research community. They are well placed to contextualize PRO results for both the health policy and the patient community, helping to frame PRO results to aid their decision making. In a study of patients with glioblastoma (see Table 2), the patient advocacy group, the American Brain Tumor Association (ABTA), was not only a co‐designer of the measure, but also supported the interpretation and publication of the findings, including reviewing abstracts and posters for scientific meetings.

The dissemination of PRO findings in the patient community represents an opportunity for patient education and awareness raising about the value of PROs in general as well as reporting on specific study findings. Communication between advocates and patients, caregivers or health‐care professionals can support and encourage the continued evolution of PROs by routinely integrating the patient perspective. During this process, advocates may consult with patients about areas related to the study findings where the patient experience is undocumented, thus identifying opportunities for further PROs. Dissemination of trial findings to patients from different ethnic and cultural backgrounds might raise awareness of PROs, research processes and the value of taking part in clinical trials, thus promoting greater inclusivity. Lastly, the patient advocate is well placed to ensure that ethical guidelines and clear recommendations are in place for dissemination through peer‐reviewed channels. Their involvement at this stage may support PRO findings and contribute further to discussions around the benefit–risk of treatment options for patients or cost effectiveness for service providers.

## 3 A FRAMEWORK FOR PATIENT AND PATIENT ADVOCATE INVOLVEMENT IN PRO DEVELOPMENT

This paper and its references contain numerous practical suggestions and resources which can further support the involvement of patients and patient advocates in PRO development, implementation and dissemination. In addition, a framework has been developed to guide a collaborative partnership between health‐care professionals and patients and patient advocates, which enables the inclusion in clinical trials of PROs that truly reflect what matters to patients.

### 3.1 Patient and patient advocate selection

At the outset of the PRO development or selection process, the optimum number of patients and patient advocates needed to make a meaningful and manageable contribution should be discussed and determined.^24^ This may be guided by requirements for validation or testing, by available resources and/or by the number of patients and/or patient advocates who are available and willing to participate. Provision for meetings, qualitative interviews, focus groups and feedback mechanisms needs to take into account patient and patient advocate numbers, where they are based geographically and how much time they are able to give to the role.^24^ A further suggestion is for patients and patient advocates to be represented on such projects in numbers proportional to health‐care professionals so they are equal partners.^24^

**Figure 2:**
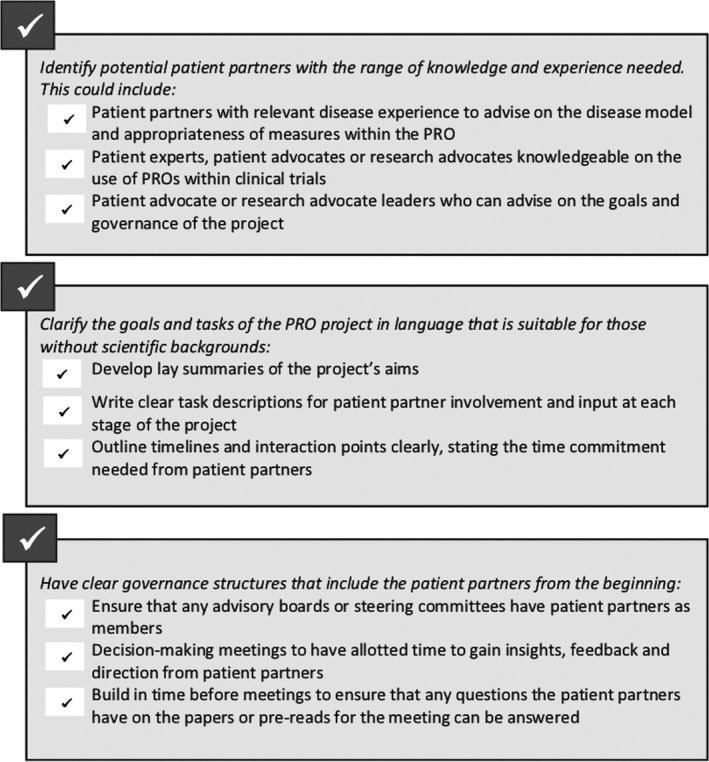
Checklist for involving patient partners when initiating a PRO development project

The criteria used for the selection of patient partner (patients, patient advocates and caregivers in some instances) will be dependent on best fit between the partner, the focus of the research and their anticipated roles in developing the PRO.^24^, ^25^, ^56^ Best‐fit patient advocacy organizations may be assessed by considering knowledge or experience of research or medical product development, size of membership, resources for supporting patients including funding opportunities or knowledge about funding.^25^ Identifying patient partners to take part in PRO development requires an understanding of the level of involvement needed for each step. Individuals with experience and knowledge of the disease, for example, may be able to contribute to a disease model. For the review and development of PRO domains, it may be useful to identify patient experts, patient advocates or indeed research advocates who already have some level of knowledge about PROs and their use in clinical trials. There are a growing number of patients who are able to develop this expertise following the introduction of specialist training courses designed to enable them to be active participants in research.^57^ A written task description defining the role of the patient partners in the project is recommended both to guide the identification of appropriate individuals and to support them in making a decision about their involvement.^22^, ^58^ In addition to outlining level of knowledge and expertise, task descriptions may also include requirements such as communication skills or being comfortable speaking in a group setting.^58^ In the identification of patient partners with the appropriate level of expertise, clinical networks and networks of patient advocacy groups can be a valuable resource.^22^, ^46^ A checklist for involving patient partners when initiating a PRO development project is shown in Figure 2.

When approaching a potential patient partner ensure that the individual understands the nature of the project, the goals of patient involvement as well as the time commitments and the expectations. Make clear that this is an invitation to take part and is not compulsory. In some cases, the patients and caregivers who have the most relevant expertise to contribute to the development of a PRO are those with a significant burden of illness. The project timeline should be structured to reflect this, allowing for flexibility and plenty of time for responses from patient partners. Project leaders may need to adjust the numbers involved to ensure the required level of patient contribution throughout the process.

### 3.2 Optimizing communication in the development team

Traditionally, the patient and patient advocate have been involved only at the later stages of giving feedback on what has already been conceived and is in development. When they are fully engaged in the development process from the very beginning, the patient and patient advocate can actively influence decisions, pose questions and even drive the process. This more comprehensive and complex approach brings with it the need for communication and facilitation skills in order to achieve meaningful and productive engagement.^24^ An open dialogue between all stakeholders that allows discussion of both the patient experience and formal care systems is crucial for mutual understanding.^22^ Also important is the support required for all team members, including regular and direct communication, use of a partnering system between clinical researchers and patients and patient advocates where appropriate and opportunities for reflection on the collaborative process.^22^

### 3.3 Supporting resources

Finding the time and financial and training resources required to administer PRO measures in today's health‐care environment may be challenging.^19^, ^59^ However, support is growing for the development and implementation of PROs and appropriate measures. The Patient‐Centred Outcomes Research Institute (PCORI) provides study funding for patient‐centred comparative clinical effectiveness research which aims to identify which health‐care options work best for the patients themselves. Funding is contingent in part on the inclusion of patients and patient advocates as partners in the research process.^60^ The PROlearn project in Birmingham, UK, offers resources which include advice for patient advocates on their involvement in PRO development and implementation.^61^ The European Patients' Academy (EUPATI) is led by a consortium from patient organizations, industry, academia and other not‐for‐profit organizations and provides training programmes and educational resources for patients to give them the skills required to understand and contribute to medicines research and development and regulatory processes.^57^

The Setting International Standards in Analyzing Patient‐Reported Outcomes and Quality of Life Endpoints Data (SISAQOL) Consortium is a multidisciplinary group which works to standardize the analysis of HRQoL and other PRO data in cancer clinical trials, including best practice on addressing missing data.^62^ The aim of this standardization is to facilitate comparison between trial findings and thus to inform clinical decision making, treatment labelling and health‐care policy.^62^ The SISAQOL recommendations (due to be published in 2019) will support PRO analysis and interpretation in the same way that the SPIRIT‐PRO and ISOQOL guidelines offer recommendations on PRO protocol content and reporting of findings respectively.^6^, ^28^ Standardized outcomes and measurement tools for several kinds of cancer which aim to focus on what matters most to the patient have been included in sets developed by the International Consortium for Health Outcomes Measurement (ICHOM).^63^ The sets are further supported by implementation guidance and case studies.^63^

Patient advocate familiarity with guidelines, organizations and resources may be helpful in ensuring that their input into the PRO development process is informed and follows best practice. It may also help advocacy organizations to pass on relevant information and training opportunities to patients engaged in PRO development.

### 3.4 A framework for moving forward

**Figure 3:**
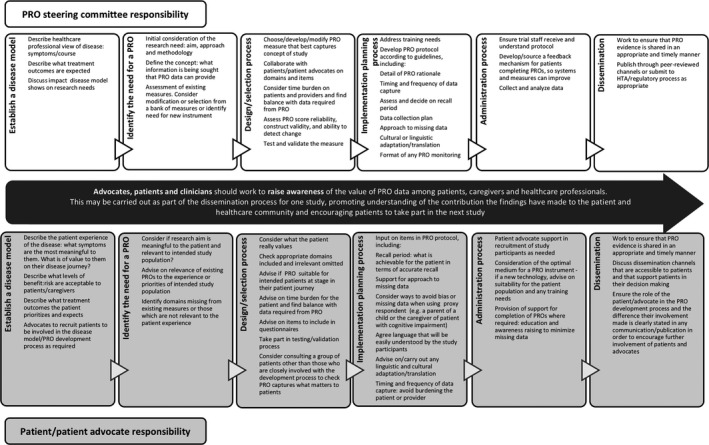
Stages of PRO development and suggested input from the PRO development steering committee and patients/patient advocates at each stage

To change the current paradigm of PRO development in cancer clinical trials, patients and patient advocacy organizations need to work with industry, academia, clinicians and other health‐care professionals, regulatory bodies, funding bodies, and health‐care professional organizations or societies. For patients and patient advocates to be active participants at all levels and from the very beginning of the PRO development, selection and implementation process, commitment to their involvement and collaborative working with these groups or individuals is essential. A flowchart showing the steps and responsibilities of patients and patient advocates and steering committee members involved in PRO development is given in Figure 3.

## 4 CONCLUSION

PROs in clinical trials are often failing to provide robust patient‐relevant data especially when suboptimal, non‐disease‐specific or outdated measures are used which do not reflect the patient reality.^17^, ^19^, ^64^ PRO data are crucial to fully informed treatment assessments and choices made by regulatory bodies, industry, policy makers, clinicians and patients.^19^, ^21^, ^65^ The involvement of patients and patient advocates may not solve all the challenges faced in developing reliable, acceptable and valid PROs, but their involvement plays an indispensable role in the development of meaningful PROs that fully realize the value of patient‐centred data in clinical trials. The patient and patient advocacy community should be working collaboratively with researchers, industry, clinicians and investigators. Patient and patient advocate input has great potential to improve design, reduce missing data and impact regulatory processes, policy decisions, shared decision making, and ultimately patient outcomes. As leaders of patient advocacy organizations, we are committed to working towards this goal and we invite all stakeholders to join us.

## CONFLICT OF INTEREST

Bonnie Addario, Marcia K Horn, and Linda U Krebs; I have no financial or other relationships, conditions, or circumstances that present a potential or actual conflict of interest that influenced what is written in the submitted work. Jan Geissler; Advisory roles and consultancy on meaningful patient engagement to Amgen, Biomarin, Bristol‐Myers Squibb, Janssen, Novartis, Pfizer, Roche, Servier, UCB. Contributions to IMI‐funded consortium projects HARMONY and EUPATI by EFPIA member companies. Deborah Maskens; I have no financial conflicts of interest except for some reimbursement of travel/accommodation costs to some essential meetings locally within Canada by associations that have obtained funding from the pharmaceutical industry. Any honoraria are donated in full to Kidney Cancer Canada (registered charity) for patient support, services and research. Kathy Oliver; I believe that I have no actual, perceived or potential conflict of interest with regard to this paper. The International Brain Tumour Alliance (IBTA) of which I am Chair and Co‐director accepts educational and non‐directed grants for its work from a number of pharmaceutical and medical device companies. IBTA also accepts a small number of donations from the general public and on occasion we have been supported by private trusts/bequests. In my role for Kathy Oliver Consulting, I have accepted honoraria for speaking engagements and, from time to time, providing advisory/consultancy services for a number of pharmaceutical and medical device companies. Ananda Plate; All funding and honoraria received are paid directly to Myeloma Patients Europe AISBL (non‐profit organization registered in Belgium) for member services and research to the benefit of the myeloma patient community. Erin Schwartz; The Max Foundation, for which I am VP of Global Engagement, is a non‐profit 501c3 entity which is independent and is a non‐governmental organization. The organization does receive funding from the pharmaceutical industry, including Novartis and other companies. In the last several years, I have participated in advisory boards with Pfizer and BMS which required consulting contracts. I am also on a high‐level committee with Novartis related to our patient access programme work. Nicole Willmarth; No conflict of interest to declare.

## Data Availability

Data sharing is not applicable to this article as no new data were created or analysed in this study.
